# Improvement and Optimization of Standards for a Preclinical Animal Test Model of Laser Induced Choroidal Neovascularization

**DOI:** 10.1371/journal.pone.0094743

**Published:** 2014-04-29

**Authors:** Yanji Zhu, Qing Lu, Jikui Shen, Ling Zhang, Yushuo Gao, Xi Shen, Bing Xie

**Affiliations:** 1 The Department of Ophthalmology, Ruijin Hospital, Shanghai Jiao Tong University School of Medicine, Shanghai, China; 2 The Departments of Ophthalmology, The Johns Hopkins University School of Medicine, Baltimore, Maryland, United States of America; The University of Melbourne, Australia

## Abstract

**Background:**

As the murine model of laser-induced choroidal neovascularization (CNV) is becoming the most established and commonly utilized model worldwide for studying the pathogenesis of CNV and its response to treatment, specific operating standards are yet to be clarified. The purpose of this study is to compare the lesion size of CNV in mice with different ages, sex, durations of CNV process, and treated positions of laser spots, to make recommendations that may improve and optimize the quality of the model.

**Methods and Results:**

C57/BL6 mice of different ages were treated with diode laser photocoagulation per eye and perfused with PBS containing fluorescein-labeled dextran at different time of observation. Choroid flat mounts, were then examined by fluorescence microscopy for the measurement of CNV area. Messenger-RNA expression levels of several angiogenic cytokines in eye cups of male and female C57BL/6 mice at 5–8 and 16–20 week-old were analyzed by real-time RT-PCR assay. The results showed significantly more CNV area in eyes of female mice compared to male mice with the expression level of several angiogenic cytokines elevated. 16–20-week-old female mice developed the biggest area of CNV. The mean area of CNV increased significantly at the 14^th^ day after photocoagulation. Laser spots delivered 1PD away from the optic disc induced the biggest area of CNV compared to those 2PD or 3PD away. Interaction of NV was observed in laser spots delivered less than 1PD away from each other.

**Conclusion:**

The current results suggest that 16–20-week-old female C57BL/6 mice developed the most distinct CNV lesion size with laser spots delivered 1PD away from the optic disc. The best time to observe and analyze is the 14^th^ day after photocoagulation.

## Introduction

Age-related macular degeneration (AMD) is a progressive degeneration process initiating in Bruch's membrane, evolving into the retinal pigment epithelial (RPE) and ultimately the overlying photoreceptors. Characterized by sub-retinal deposits (drusen) with or without evidence of damage to underlying RPE, AMD is responsible for the majority of blindness among individuals older than 65 years in the industrialized world [1]. Early stage of AMD is often called the “dry” form of the disease, which is a more common and milder form of AMD, accounting for 85% to 90% of all cases. As the small hard drusen enlarges with age, RPE cells begin to lose and eventually the overlying photoreceptors degenerate. Pathogenetic mechanisms of inflammation, oxidative damage, and RPE senescence play a central role in this process [2]. In the minority of cases, abnormal blood vessels (choroidal neovascularization, CNV) protrude from the choroid through Bruch's membrane towards the retina, called “wet” AMD [3], thus leaking fluid and blood into the tissue at the back of the eye, causing an acute loss of central vision.

CNV, a dynamic process with initiation, maintenance and involution stages, is a common pathological process of numerous chorioretinal diseases [4]. It contributes to the severe vision loss especially in patients with AMD, as well as pathologic myopia [5], presumed ocular histoplasmosis syndrome [6], angioid streaks [7], and idiopathic polypoidal vasculopathy[8]. In all of these conditions, a break in Bruch's membrane is necessary for the development of CNV, enabling the growth of new blood vessels into the sub-retinal space and initiating the evolution of CNV. The break in Bruch's membrane can be induced by laser, surgery or in the setting of transgenic mice. When the break occurs, it makes possible that inflammatory, angiogenic and extracellular matrix components such as choriocapillary endothelial cells, pericytes and inflammatory cells come into the sub-retinal spaces. Angiogenic cytokines as vascular endothelial growth factor (VEGF), as well as a member of its family, placental growth factor (PlGF), play a pivotal role in inducing proliferation and recruiting pericytes during angiogenesis [9],[10]. VEGF, together with inducible nitric oxide synthase (iNOS), increased vascular permeability. Angiopoietin2 plays a facilitative role at sites of vascular remodeling to revert the vessels to a more plastic and unstable state [11]. Thus CNV takes place. As the cytokine production decreases associated with scarring and fibrosis, CNV begins to decline [12].

The laser-induced animal model was firstly employed on monkeys to show an experimental model of CNV in 1979 [13], and was later successfully used to develop photodynamic therapy and anti-VEGF therapy [14], [15]. In 1998, Tobe et al. applied this model to mice and developed the murine model of laser-induced CNV for the first time [16]. Featuring such advantages as appropriate time course of events (1–2 weeks), high reliability and cost-effectiveness, murine model of laser-induced CNV is becoming the most established and commonly utilized model worldwide for studying the pathogenesis of CNV and its response to treatment [16], [17]. However, several factors affect the results of the quantification of the CNV lesion. For instance, different ages, sex and durations of CNV process result in varied area of CNV. Therefore, we compared the lesion size of CNV in murine model of laser-induced CNV in order and made recommendations that may improve and optimize the quality of the model.

## Materials and Methods

### 1. Mice and Ethics Statement

All mice used in this study were pathogen-free C57BL/6 mice. All procedures and animal care were performed in accordance with the National Institutes of Health *Guide for the Care and Use of Laboratory Animals* with the approval (SYXK-2003-0026) of the Scientific Investigation Board of Shanghai Jiao Tong University School of Medicine, Shanghai, China. All efforts were made to minimize suffering.

### 2. Mice Model of CNV

CNV was induced by laser photocoagulation with rupture of Bruch's membrane as previously described [16]. Briefly, mice at the age of 5–8, 16–20 weeks old and female mice at 30–40 weeks old were anesthetized with ketamine hydrochloride (100 mg/kg body weight), and pupils were dilated with 1% tropicamide. Burns (100 µm spot size, 0.1 second duration, 100 mW) were then performed in 3, 12 and 9 o'clock positions in the retinas with distance of less than 1 papillary diameter (PD), 1PD, 2PD, or 3PD away from the optic disc. The photocoagulation process was delivered by a system of an OcuLight GL diode laser (Iridex, Mountain View, CA, USA), with a cover slide placed on the cornea as a contact lens to view the retina. Disruption of the Bruch's membrane was identified by a bubble at the site of laser impact. Thus, only burns that were produced with a bubble at the time of laser were included in this study.

### 3. Fluorescence Perfusion and Choroidal Flat Mounts Analysis

5,7,9,11,14,17,19 or 21days after photocoagulation, the mice were perfused with 1 ml of PBS containing 50 mg/ml of fluorescein-labeled dextran (2×10^6^ average molecular weight; Sigma-Aldrich, St. Louis, MO, USA) from the left ventricular, and euthanized 5 minutes later. Eyes were then enucleated and fixed in 4% formalin for 5 hours. Choroidal membranes were carefully dissected, flat-mounted, and examined by fluorescence microscopy. Images were captured with a digital still camera (Nikon Instruments Inc., New York, NY). Image analysis software (Image-Pro Plus; Media Cybernetics, Silver Spring, MD, USA) was used to measure the total area of CNV at each rupture site, with the investigator masked with respect to the experimental groups.

### 4. Real-Time RT-PCR

**Figure 1:**
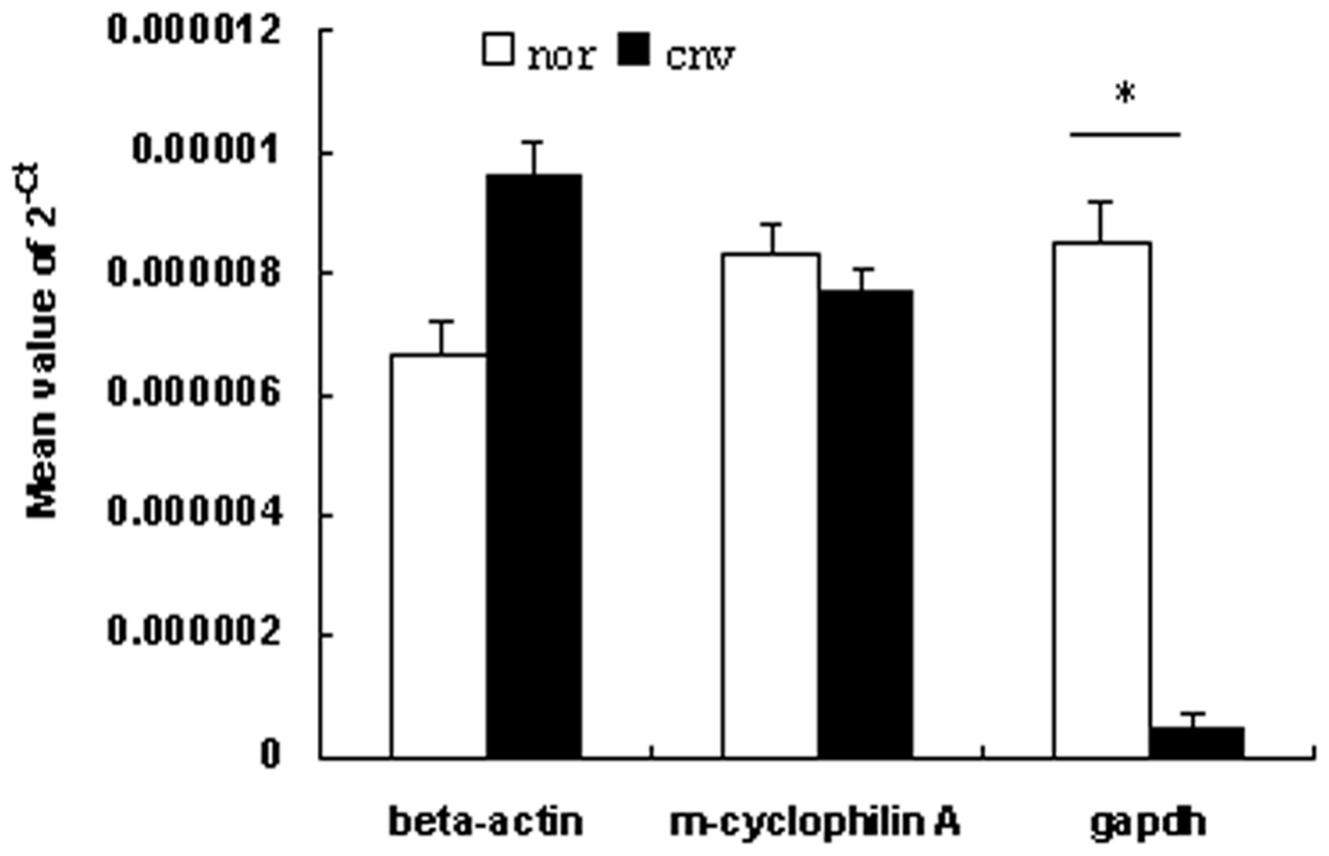
Validation of endogenous control genes in lasered and unlasered mice eyes. 5–8-week-old female C57BL/6 mice were prepared for validation of such endogenous control genes as m-cyclophilin A, beta-actin and gapdh. Three days after photocoagulation at 10 locations per eye, the mRNA expression levels of these three housekeeping genes in eyecups were compared with those of age-matched unlasered eyes. In order to determine whether the expression of the potential internal control gene varies under the experimental conditions, the realtime PCR data was presented from the replicate cDNAs as 2^−Ct^ and Student's t-tests were performed between the treated and untreated groups as described [9]. The fold changes in the three genes in the treated samples compared to the untreated samples were 1.44 for β-actin, 0.94 for cyclophilin A, and 0.06 for gapdh. Student's t-tests showed that there was significant difference between the treated and untreated samples in gapdh expression (P<0.01), but none in β-actin (P = 0.14) or cyclophilin A (P = 0.93). This suggests that cyclophilin A is presumably the most suitable endogenous control among these three genes.

To further quantitatively analyze the mRNA expressions in eye tissues, real-time RT-PCR assays were performed. Male and female C57BL/6 mice aged 5 to 8 and 16–20 week-old were prepared for analysis of the expressions of VEGF, PlGF, iNOS and Ang2, and 5–8-week-old female mice were subjected to the validation of such endogenous control genes as m-cyclophilin A, beta-actin and glyceraldehyde-3-phosphate dehydrogenase (Gapdh). The mRNA expressions were quantified in laser-induced CNV mouse model and age matched controls. Briefly, mice were subjected to laser-induced rupture of Bruch's membrane at 10 locations in each eye. Three days after laser-treatment, as the expression of angiogenic cytokines and molecules reaches its peak [18], mice were euthanized and eyes were enucleated. The anterior segments were removed from each eye after an annular incision along the limbus, and the RNA was isolated from eye cups (retina-RPE/choroid-sclera complex) using Trizol reagent (Invitrogen) in accordance with the manufacturer's instructions [19]. Age-matched untreated mice were employed as controls. 2 µg of each sample of total RNA, pretreated with DNase I (Promega), was reverse-transcribed into complementary DNA (cDNA) using M-MLV Transcriptase and oligo dT Primers (Promega), according to the manufacturer's instructions. Quantitative RT-PCR analyses were performed as described previously [20]. Each PCR was carried out in a 20 µl volume using iQ SYBR Green Supermix (Bio-Rad) for 10 minutes at 95°C denaturation, followed by 40 cycles at 95°C for 30 seconds and 60°C for 1 minute in ABI7500. To determine whether the expression of the potential internal control gene varies under the experimental conditions, the realtime PCR data was presented from the replicate cDNAs as 2^−Ct^ as described [21]. To normalize for differences in efficiency of sample extraction or cDNA synthesis by reverse transcriptase, we use the most stable endogenous control verified, m-cyclophilin A, as a housekeeping gene (Fig. 1). The ΔΔCT method was used for relative quantification. Primers used were: cyclophilin A forward 5′-CAGACGCCACTGTCGCTTT-3′ and reverse 5′-TGTCTTTGGAACTTTGTCTGCAA-3′; PlGF forward 5′-TTGGCGACCATGTCAGAACTTTGC-3′ and reverse 5′ TGGCCAAGGATCTTCATGTCCTGT-3′; VEGF forward 5′-TTCATAAGGCGTGGCATACA-3′ and reverse 5′-TCACCCAGGAGGACATCTTA-3′; iNOS forward 5′-CCCTTCAATGGTTGGTACATGG-3′ and reverse 5′-ACATTGATCTCCGTGACAGCC-3′; Ang2 forward 5′-CACCACTTGCACACACAAAG-3′ and reverse 5′-AGCCATTCTCACAGCCAATAA-3′; gapdh forward 5′- AACAGCAACTCCCACTCTTC-3′ and reverse 5′- CCTGTTGCTGTAGCCGTATT-3′; beta-actin forward 5′- CATCCGTAAAGACCTCTATGCCAAC-3′ and reverse 5′- ATGGAGCCACCGATCCACA-3′. Two eye cups of each mouse were considered as one sample. (n = 8 mice/group).

### 5. Statistics

Data were expressed as mean±SEM. Statistical significance was analyzed by t-test or one-way or two-way ANOVA with Student-Newman-Keuls method for multiple comparisons using SAS 9.0 software. P<0.05 was considered statistically significant.

## Results

### 1. Eyes of Older Female Mice Showed Significantly More Distinct CNV

**Figure 2:**
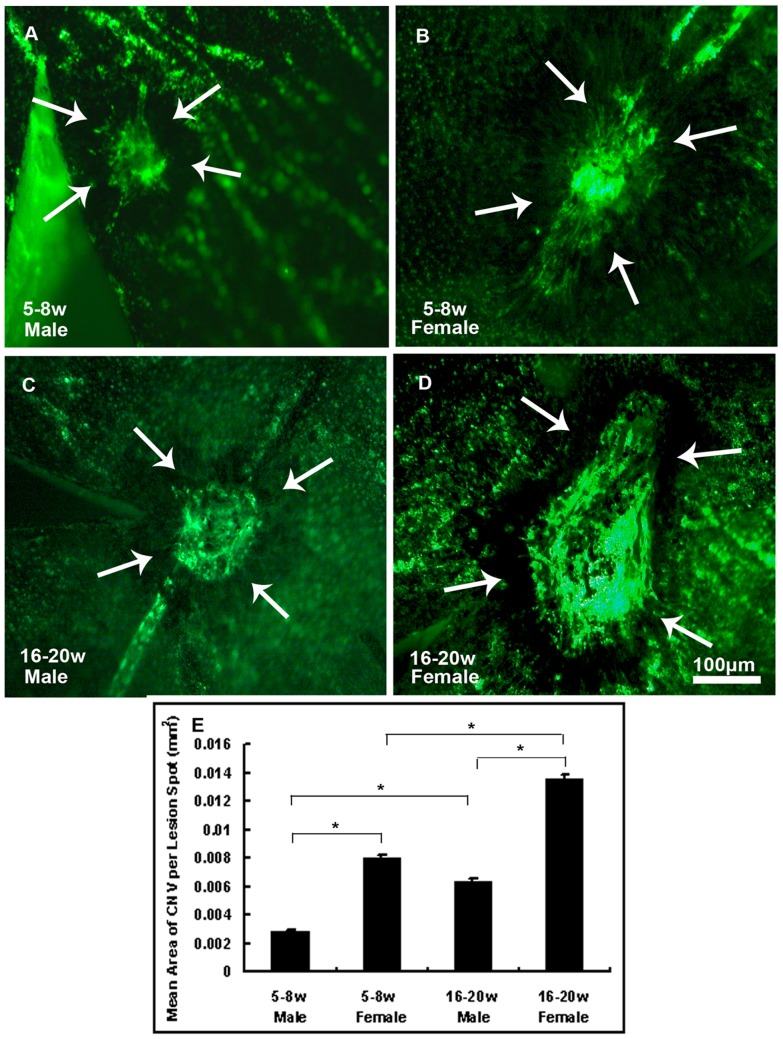
Significantly bigger area of CNV was observed in older female mice. 5–8-week old male (Fig. 2A) or female (Fig. 2B) and 16–20-week old male (Fig. 2C) or female (Fig. 2D) C57BL/6 mice were anesthetized and burns were given at 3, 12 and 9 o'clock positions that were 1PD away from the optic disc in the posterior pole of retinas with a cover slide placed on the cornea as a contact lens. After 2 weeks, mice were perfused with fluorescein-labeled dextran and choroidal flat mounts were examined by fluorescence microscopy. Image analysis software (Image-Pro Plus) was used to measure the total area of CNV at each rupture site and the mean area of CNV per eye was calculated to give a single experimental value. The bars show the mean +− SEM for each group calculated from experimental values (Fig. 2E). Statistics were analyzed by two-way ANOVA. (n = 6mice/group, *P<0.001).

Male and female mice at 5–8 (Fig. 2A and 2B) and 16–20 weeks old (Fig. 2C and 2D) were prepared for CNV model. After examination of choroidal flat mounts with fluorescence microscopy, images were analyzed by Image Pro Plus to measure the total area of CNV of each rupture site. Data were shown in Fig. 2E. The bars show the mean±SEM for each group calculated from experimental values. Statistical analysis by two-way ANOVA demonstrated significant effect of age (P<0.001) and sex (P<0.001). It showed more distinct lesion size in female mice compared to males. Significant bigger CNV area was observed in older mice than in younger ones. Older female mice developed significantly the biggest lesion size. (*P<0.001)

### 2. Messenger-RNA Expressions of Several Cytokines in Lasered or Unlasered Mouse Eyes Showed Association with Gender and Age

**Figure 3:**
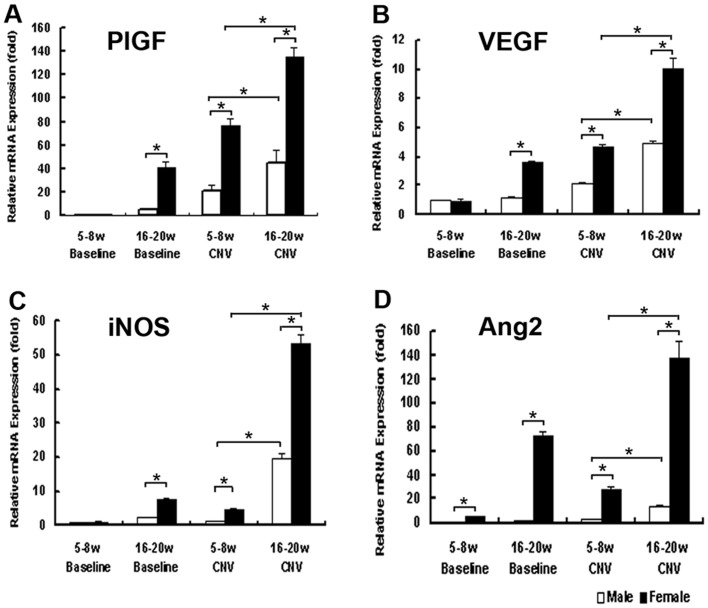
mRNA expression of several angiogenic cytokines in age-matched lasered or unlasered mice eyes. mRNA expression of PlGF (A), VEGF (B), iNOS (C) and Ang2 (D) were evaluated in both male and female mice at 5–8 and 16–20 weeks old with realtime RT-PCR assay. CNV mice were subjected to laser induced photocoagulation at 10 positions within 1 to 2 PD away from the optic disc. Age-matched untreated ones were employed as controls. Three days after photocoagulation, real-time RT-PCR assays were performed. Two-way ANOVA analysis suggested both significant effect of age and sex on the expression levels of these genes. It was demonstrated that significantly increased expression of these mRNAs in female mice compared to age matched males and baselines (P<0.01), and older mice expressed significantly higher levels of these cytokines than young ones with the same gender (P<0.01). The expression levels were expressed as n-fold increases from baseline eyes of male mice at 5–8 weeks old. The bars show mean +− SEM. Statistics was analyzed by two-way ANOVA. (n = 8mice/group, *P<0.01).

Real-time RT-PCR assays were applied to eyes of both male and female mice at 5–8 and 16–20 week-old in order to investigate the involvement of some cytokines associated with CNV formation. Two-way ANOVA analysis suggested significant effect of age (P<0.01) and sex (P<0.01) on the expression levels of these genes. In good accordance with previous studies [22], [23], 3 days after laser photocoagulation, significantly increased expressions of such cytokines as PlGF (Fig. 3A), VEGF (Fig. 3B), iNOS (Fig. 3C) and Ang2 (Fig. 3D) were observed in females compared to age matched males, indicating sex hormones play a role in modulation of gene expression in CNV process. Furthermore, 16–20-week-old mice were demonstrated to express significantly higher levels of these cytokines compared to 5–8-week-old ones, indicating a dual regulation of these cytokines by hormones and age. There also showed significant elevation of iNOS and Ang2 in older females compared to those of males, indicating these two cytokines were influenced more by sex hormones than by age. (*P<0.01)

### 3. 16 to 20-week-old Female Mice Developed the Biggest Mean Area of CNV

**Figure 4:**
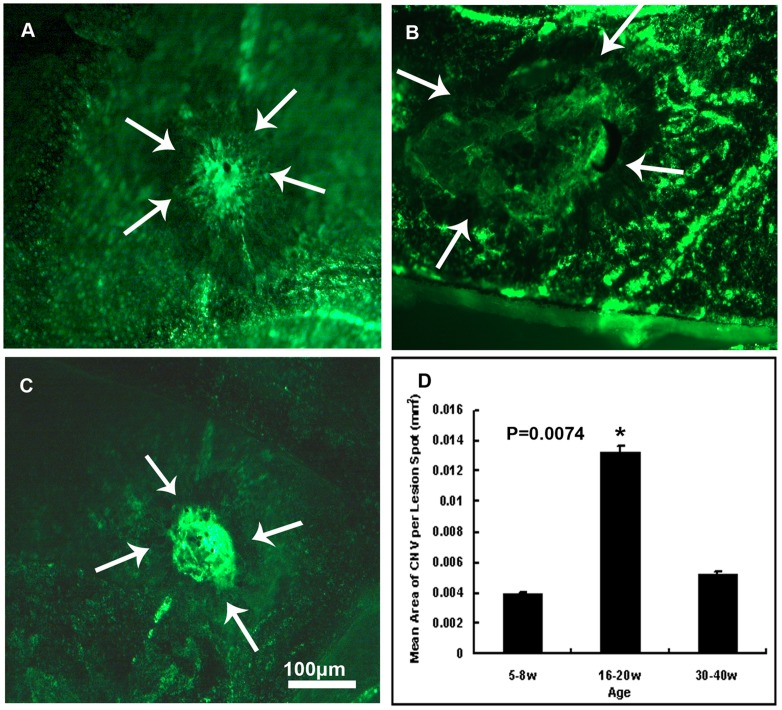
Mean area of CNV in mice of different ages. Statistical significance of CNV area was detected among mice aged 16–20 weeks, 5–8 weeks and 30–40 weeks (P = 0.0074). There seemed to be significantly the biggest CNV area in mice aged 16–20 weeks compared with mice aged 5–8 weeks and 30–40 weeks. Female mice aged 5–8 (Fig. 4A), 16–20 (Fig. 4B) or 30–40 (Fig. 4C) weeks were given laser photocoagulation spots at 3, 12 and 9 o'clock positions 1PD away from the optic disc in the posterior pole of retina of each eye with a cover slide placed on the cornea as a contact lens. After 2 weeks, mice were perfused with fluorescein-labeled dextran and choroidal flat mounts were examined by fluorescence microscopy. The area of CNV of each spot was measured by image analysis and data were analyzed by one-way ANOVA and expressed as mean +− SEM, as was shown by the bars (Fig. 4D). Mice aged 16–20 weeks showed significantly biggest mean area of CNV compared with mice aged 5–8 weeks and 30–40 weeks (P = 0.0074). But there showed no statistical significance of mean area of CNV between 5–8 week-old and 30–40 week-old mice. (n = 7mice/group).

Female mice aged 5–8 w, 16–20 w, and 30–40 w were prepared for CNV model as described previously. Choroid membranes were flat mounted and images were analyzed after 2 weeks. Fig. 4A, 4B, 4C showed flat mounts of choroidal membranes of mice aged 5–8 ,16–20, 30–40 weeks under fluorescence microscopy at 100× magnification, respectively. Data were analyzed by one-way ANOVA and expressed in Fig. 4D. It showed significant difference of CNV area among these three groups (P = 0.0074). Mice aged 16–20 weeks old developed the significantly biggest mean area of CNV (0.0133±0.0005 mm^2^) compared to those aged 5–8 (0.0040±0.0008 mm^2^) and 30–40 (0.0052±0.0001 mm^2^) weeks old, but no statistical significance was detected between mice aged 5–8-week and 30–40-week old.

### 4. The Mean Area of CNV Significantly Increased at Day14 after Laser Photocoagulation

**Figure 5:**
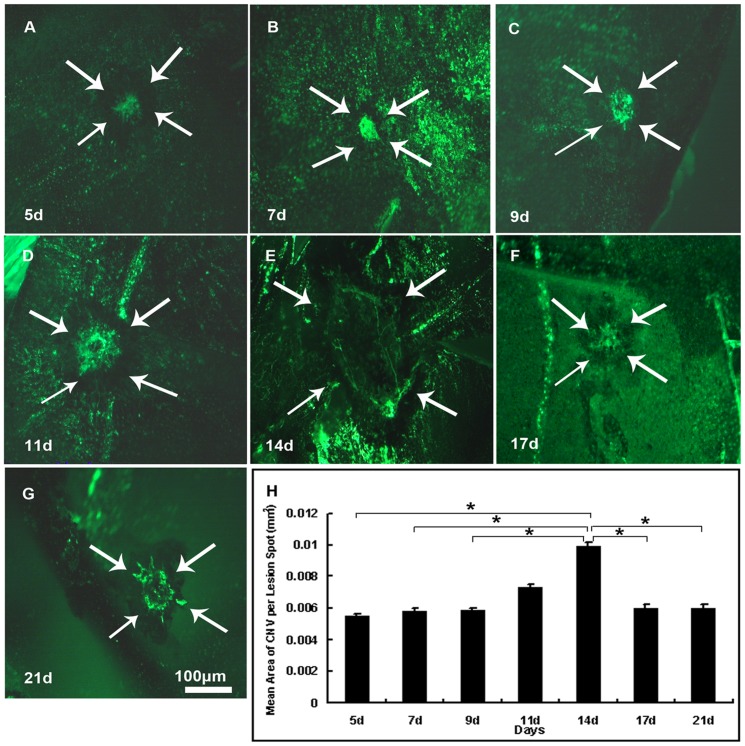
Mean area of CNV significantly increased at day 14 after photocoagulation. 16–20 week-old female C57BL/6 mice were given laser photocoagulation spots at 3, 12 and 9 o'clock positions 1PD away from the optic disc in the posterior pole of retina of each eye with a cover slide placed on the cornea as a contact lens. After 5, 7, 9, 11, 14, 17, 21 days, mice were perfused with fluorescein-labeled dextran and choroidal flat mounts were examined by fluorescence microscopy. The mean (±SEM) area of CNV was calculated from each experimental value of each laser spot. CNV appeared from day 5 (Fig. 5A), increased significantly at day 14 (Fig. 5E, P = 0.017), and decreased thereafter. Significant differences were observed of day 14 from any other point except for day11, but no significant difference was detected between day11 and any other day point Figure 5A to 5G show choroidal representative flat mounts of mice euthanized 5, 9, 11, 14, 17, 21 days after photocoagulation at 100× magnification. The laser spots are shown by arrows. Statistics were analyzed by one-way ANOVA with Student-Newman-Keuls method for multiple comparisons. (n = 6mice/group, *P<0.05).

16 to 20-week-old female mice were applied to laser photocoagulation for CNV model. Images of choroidal membrane flat mounts were analyzed 5 (Fig. 5A), 7(Fig. 5B), 9 (Fig. 5C), 11(Fig. 5D), 14 (Fig. 5E), 17 (Fig. 5F) and 21 (Fig. 5G) days after photocoagulation, and data were analyzed by one-way ANOVA and shown in Fig. 5H. A significant increase was observed of mean area of CNV at day14 (P = 0.017), but decreased thereafter. Multiple comparisons with Student-Newman-Keuls method indicated significant differences of day 14 from any other point except for day11, but no significant difference was detected between day11 and any other day point.(*P<0.05)

### 5. Laser Spots Delivered 1PD Away from the Optic Disc Induced the Biggest Area of CNV

**Figure 6:**
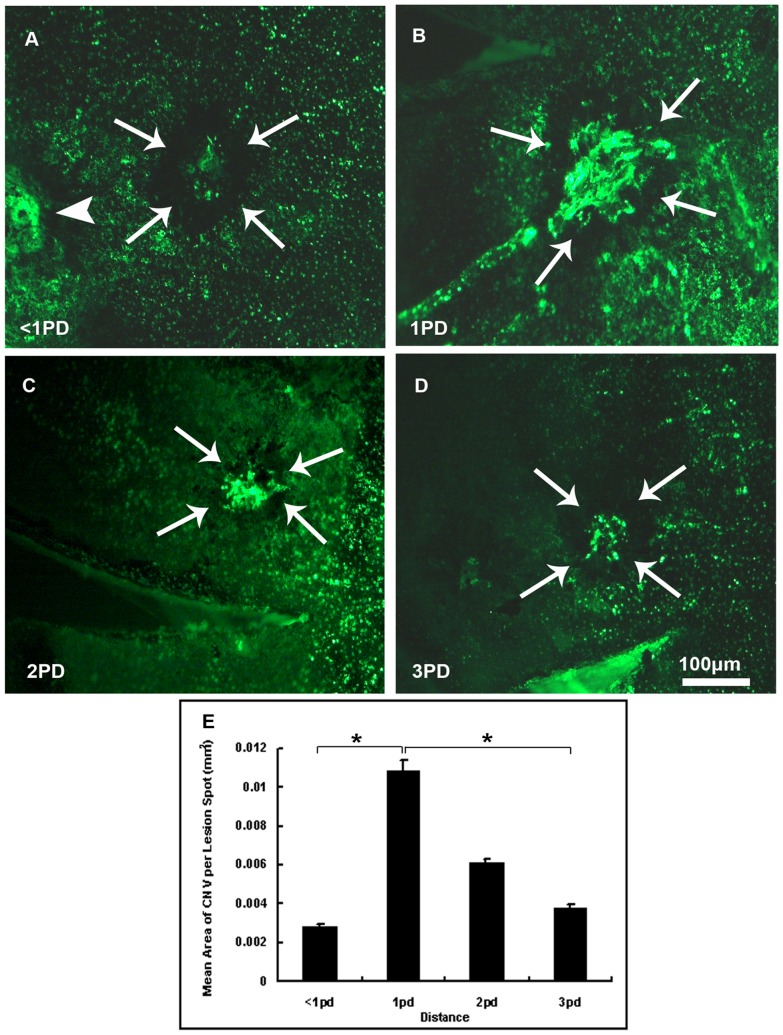
Mean area of CNV of laser spots delivered with different distances away from the optic disc. CNV area of spots delivered with distance of <1PD, 1PD 2PD and 3PD away from optic disc was examined.16–20 week-old female C57BL/6 mice were given laser photocoagulation spots at 3, 12 and 9 o'clock positions in the retinas with distance of <1PD (A), 1PD (B), 2PD (C), or 3PD (D) away from the optic disc of each eye with a cover slide placed on the cornea as a contact lens. After two weeks, eyes were enucleated after perfusion with fluorescein-labeled dextran and choroidal flat mounts were examined by fluorescence microscopy. The area of CNV of each spot was measured by image analysis and the mean (±SEM) area of CNV of each group was calculated with the investigator masked with respect to the experimental groups. Significant difference was observed among these four groups. It was shown that significantly more CNV area (0.0109±0.0005 mm^2^) appeared in spots 1PD away from the optic disc (P = 0.008), while 0.0028±0.0002 mm^2^ in spots <1PD (arrow head showed the adjacent optic disc in Figure 6A)away from the optic disc and 0.0038±0.0001 mm^2^ in spots 3PD away from the optic disc (Fig. 6E). The mean CNV area tended to decrease as spots became farther away from the optic disc. But there was no significant difference of mean CNV area between 2PD-away spots and 1PD, <1PD, or 3PD-away spots. Statistical significance was analyzed by one-way ANOVA. The arrow head shows the optic disc. (n = 5mice/group, *P<0.01).

At least three laser spots were delivered in eyes of 16 to 20-week-old female mice at 3, 12 and 9 o'clock positions in the retinas with distance of <1PD, 1PD, 2PD and 3PD away from the optic disc. Two weeks later, eyes were enucleated after perfusion and choroidal flat mounts were examined by fluorescence microscopy. Fig. 6A to 6D showed CNV induced by spots delivered <1PD, 1PD, 2PD, 3PD away from the optic disc at 100× magnification under fluorescence microscopy respectively. Statistical significance was detected among spots delivered 1PD away from the optic disc and the others with one-way ANOVA analysis (P = 0.008),. As is shown from the bars (Fig. 6E), laser spots delivered 1PD away from the optic disc induced biggest area of CNV (0.0109±0.0005 mm^2^) compared with spots <1PD, 2PD or 3PD away from the optic disc though there showed no significant difference between 2PD-away spots and 1PD, <1PD or 3PD away spots by Student-Newman-Keuls test. (*P<0.01)

### 6. Interaction of NV Was Observed in Laser Spots Delivered Less Than 1PD Away from Each Other

**Figure 7:**
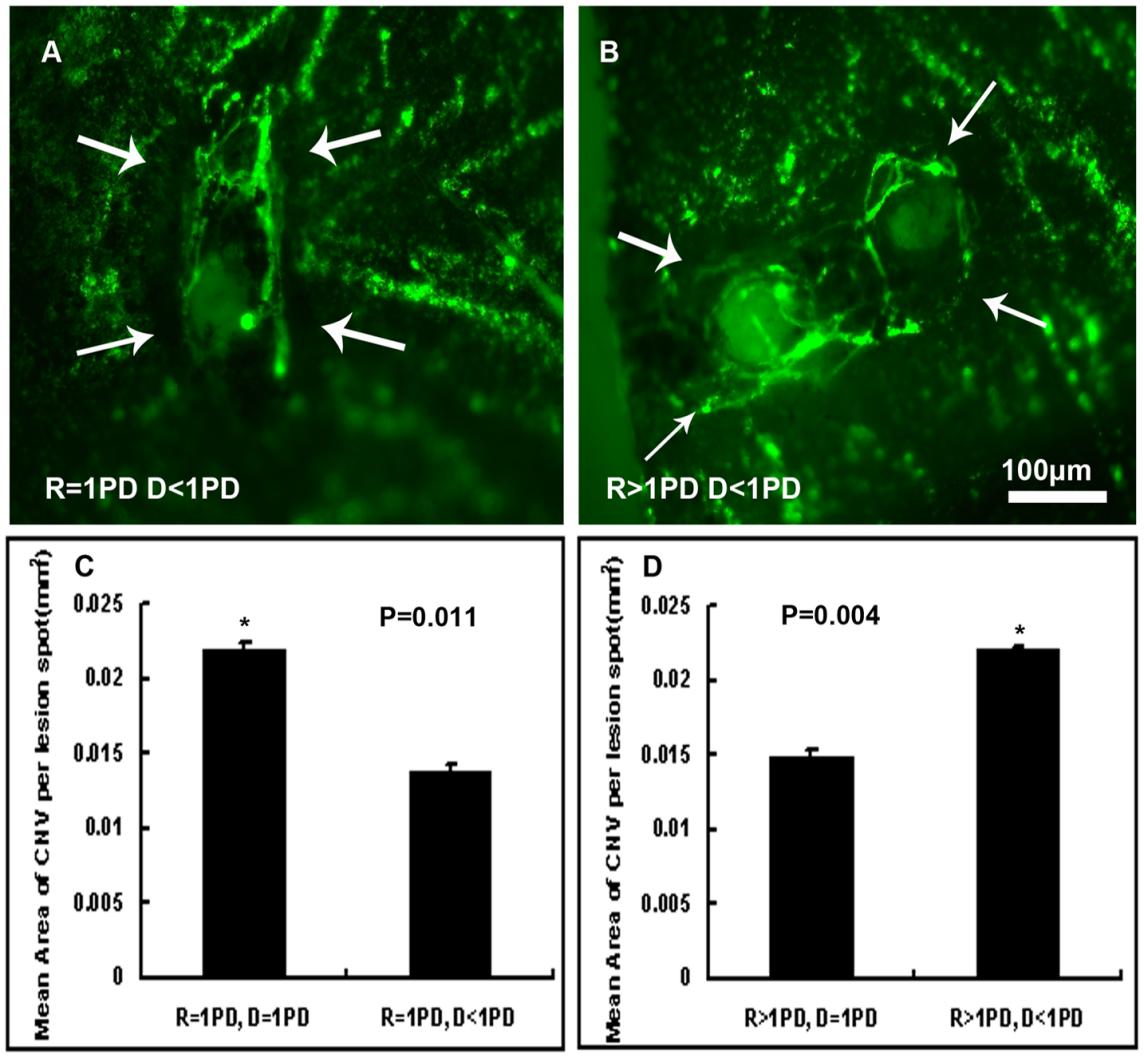
Interaction of NV observed in laser spots delivered further away from the optic disc and closer together. 16–20- week-old female C57BL/6 mice were given laser photocoagulation spots in the retinas with distance of 1PD from the optic disc and less than 1PD from each other, or 1PD from each other, as well as with distance more than 1PD from the optic disc and 1PD from each other, or less than 1PD from each other of each eye with a cover slide placed on the cornea as a contact lens. After two weeks, mice were perfused with fluorescein-labeled dextran and choroidal flat mounts were examined by fluorescence microscopy and the area of CNV was measured by image analysis. Each CNV lesion area was calculated as a single experimental value so as to investigate whether the distance between two adjacent laser spots would affect the area of CNV induced by photocoagulation. Statistics analyzed by student's t-test suggested interactions occurred when spots were given further away (1PD away from the optic disc) and closer together (less than 1PD from each other; n = 5mice/group; R: distance from laser spots to optic disc, D: distance between the two adjacent laser spots).

The mean area of CNV induced by laser spots with different distance away from the optic disc as well as from each other was measured and statistics were shown in Fig. 7C and 7D. In order to investigate whether distance would affect the area of CNV, each CNV lesion area was calculated as a single experiment value. Taking into consideration a normal lesion size is in the order of 0.014 mm^2^ (Fig. 4D), the results indicated interactions occurred when spots were given further away, 1PD away from the optic disc, and closer together, less than 1PD from each other.

## Discussion

The actual cause of AMD is yet to be investigated but involves ages, genetic predisposition, and environmental factors. Established epidemiologic risk factors include cigarette smoking, diet, female sex, Caucasian race, and a family history of AMD [24]–[26].

Thanks to preclinical and clinical studies, researchers have demonstrated that a few of the molecular and cellular players recruited in the microvascular microenvironment of CNV. The three interrelated systems of inflammation, angiogenesis and proteolysis are enrolled in the formation of CNV. The successful translation of angiogenesis inhibitors to clinical application depends partly on the transfer of expertise from scientists who are familiar with the biology of angiogenesis to clinicians. It is desirable that animal models of CNV be efficient and reproducible, stable and sustainable over time, exhibit similar pathologic manifestations and growth patterns to human CNV [27], be inexpensive to produce and easy to be followed. Although imperfect, animal models have led to the development of anti-angiogenic molecules in clinical practice. For example, Avastin (bevacizumab) in the treatment of colon cancer and then in the treatment of AMD and other conditions with Lucentis (ranibizumab) as well where pathological angiogenesis is implied [28], [29].

Firstly developed by Tobe et al. in 1998 [16], the murine model of laser-induced CNV has been utilized for studying the pathogenesis of CNV and its response to treatment. Yet no recognized standards about specifications of this model have been recommended so far. Since AMD is a multifactorial disease, differences of age, sex and duration of the process should be taken into consideration to ensure the quality of the model.

Our study discovered the differences of CNV lesion size with age, sex, duration of the disease process and locations of laser spots. We found that 16 to 20-week-old female C57/BL6 mice developed the most extensive CNV two weeks after photocoagulation.

Both our histological and mRNA expression assays suggested that female mice were more susceptible to CNV than male mice, which might be related to the expression level of estrogen. Epidemiological studies of human indicate a higher prevalence of AMD in women than in men [30],[31],[32]. Females may have a higher risk of AMD and the significant decrease in early AMD with increasing years from menarche to menopause supports the concept that a shorter duration of oestrogen production may increase the risk of AMD [33], and expression of estrogen receptors in surgical excised CNV in highly myopic eyes, suggesting that estrogen has important functions in the formation of myopic CNV [34]. Animal experiments showed that ovariectomy in adult mice did not increase the severity of CNV, but paradoxically estrogen supplementation after ovariectomy did increase CNV severity. It was also interpreted that estrogen supplementation with the absence of an intact ovary induced vascular smooth muscle cell proliferation and up-regulated macrophage mediators (e.g. TNF-α), which in turn results in more severe CNV [35], [36]. In vivo studies have been carried out indicating the up-regulated pro-angiogenic activity of endothelial cells as well as vascular smooth muscle cells mediated by estrogen [37]. Estrogens have also been proposed to facilitate myocardial and cerebral angiogenesis and collateral vessel formation in models of ischemia-induced NV [38]. Additionally, anti-angiogenesis is one of the actions of estrogen receptor antagonist to confront tumor angiogenesis induced by estrogen in breast cancer [39]. Furthermore, significantly faster wound healing was reported in woman than in men in clinical studies [40]. Histological studies showed that CNV represents wound healing tissue quite similar to the granulation tissue of skin wounds [41]. Growth factors involved in CNV are similar to those involved in wound healing of the skin [42]. Researches have demonstrated that estrogen promotes wound healing in both human and animal models by altering cytokine profiles and modulating the balance between matrix synthesis and degradation [43].It has been reported that estrogen enhances the production of bFGF and TGF-βin fibroblasts and VEGF production in macrophages, leading to the acceleration of wound re-innervation, re-epithelialization and granulation tissue formation [44]. Although the effects of estrogens on physiologic or pathologic neovascularization remain to be thoroughly evaluated, the existing data generally suggest that estrogen is pro-angiogenic [38], [45], which is consistent with the data we obtained.

Aging is the required risk factor for AMD, which implicates the multitude of cellular changes that accompany normal aging in the pathogenesis of AMD. There exists a balance between pro-angiogenic and anti-angiogenic factors in normal eyes. Factors disturbing the balance must occur to allow the onset of CNV, such as oxidative stress, a decrease in angiogenesis inhibitors and anatomical factors to damage the Bruch's membrane [42]. With the progressive degeneration of RPE and photoreceptors, colloid materials such as lipids, phospholipids and collagen accumulated and deposited outside the RPE, thus thickening the Bruch' membrane and forming the drusen accompanied with macular degenerative lesions. These aging changes in Bruch's membrane may cause hypoxia in the outer retina and decreased oxygen diffusion [46]., resulting in an over-expression of growth factors by RPE cells and a pro-inflammatory state., which gives rise to CNV [47]. Our study also found a higher expression level of growth factors in aged eyes than young ones, which might shift the balance to a pro-angiogenic state and tending to cause more severe CNV lesions in senile eyes.

Our study demonstrated that 16 to 20-week-old female mice developed bigger CNV lesion size than those aged 5–8 weeks and 30–40 weeks, which showed discrepancy with the study of Dot C et al. [48]. They found that older mice (one-year old) demonstrated more extensive CNV formation and a slower pace of regression than younger ones (10-week-old) by comparing CNV area of mice aged 4 weeks, 6 weeks, 10–12 weeks and one-year old at different time points. Their flat mounts analysis showed that CNV area difference was statistically significant at day7 and month8 after photocoagulation, though no statistical significance was found at day 14. We think the different ages of mice and time points we applied could partially account for the difference. First, the oldest mice we used were 30–40-week-old, which is not as old as those of Dot c. et al. It has been confirmed that aging is associated with a gradual decrease in healing capacity [41], [49]. While laser induced photocoagulation was utilized to establish the mice model of CNV, it caused local surgical trauma to the eye tissue. Delayed reepithelialisation and NV, increased macrophage populations and impaired fibroblast migration are features of wound healing in the elderly [50], [44]. Yuxi et al. demonstrated that the formation of acellular capillaries was significantly increased from 9 months, with the significant reduced expression of growth factors in vessel survival up to about 60% at 9 month (VEGF, Ang-1 and PDGF-B), which means age-dependent vessel changes are in progress.[51]. In this regard, the relative small CNV area developed in 30–40-week-old mice, compared to those of 16–20-week-old mice, could be due to age-dependent vessel changes. However, further studies of mice between 16–20-week-old and 1-year-old are required if one would like to explore the influence of age on CNV. Second, Dot C et al. discovered statistically significant difference of mean CNV area from day 7 to month 8 between 10-week-old and one-year-old mice, illustrating aging slows down CNV regression process. Previous researches also found the attenuated angiogenic response differed with age, though growth factors increased in the collagen at the early phase of wound healing and facilitated NV by promoting cell migration, accumulation and proliferation [43], [52]. Swift ME and Feng Y demonstrated that a significant reduction of endothelial responsiveness may possibly account for the delayed wound angiogenesis in aged mice [50], [53]. As a result, in other words, the decreased CNV lesion size of 30–40-week-old mice at day 14 could be attributed to a delayed response, which means the angiogenic peak of 30–40-week-old mice may come after that of .16–20-week-old ones. Thus, further studies covering a longer time course of observation after laser photocoagulation needed to be done to follow a dynamic evolution of CNV progression.

We discovered that CNV lesion size increased significantly at day 14 and decreased thereafter. This is consistent with previous studies. Experimental CNV was identified clearly by FA on day 4 and day 7. From day 14 to day 21, SD-OCT slices and HE-stained histological sections showed RPE starting covering the CNV from the rim of the lesion, which contributed to the decline of CNV [54]. The tendency to spontaneous RPE recovery and CNV regression constitutes a major limitation of the model for interventional studies [55]. Considering the physiology of CNV process in mice and the time course of events, the best time to observe and analyze is the 14^th^ day after photocoagulation.

As to the location of laser spots delivered to induce CNV, we found that laser spots delivered 1PD away from the optic disc induced the biggest area of CNV compared to those 2PD or 3PD away. It showed mutual interactions when spots were given 1PD away from the optic disc and closer together (less than 1PD). The exact mechanisms remained to be investigated, but the results indicated that laser burns should be performed in different quadrants of the retina to avoid interference of each other.

However, there is still weakness in the current study that improvements could be made. For example, we isolated mRNA from the whole posterior eyecups for the qpcr assays. Since gene expression is modulated and CNV pathologic processes enroll RPE cells, endothelial cells, astrocytes and Muller cells, it is better to pool different tissues separately (e.g. retina, RPE, choroid or sclera) so as to provide tissue-specific information about CNV mechanisms. Secondly, animals for gene expression analysis received 10 laser spots as indicated by Doc C et al. [48], rather than 3 for other parts of our study. Although all groups of animals for qpcr assay were treated the same way, it was possible to amplify the molecular process, which impaired its correlation with CNV morphologic data. Furthermore, day 3 was the only time point for gene expression analysis and prior to morphologic assessment in our study though it is peak point for proangiogenic factors [56]–[57]. However, CNV is a dynamic process as can be seen from our time course lesion data (from day 5 to day 21), and thus a more impeccable experiment design is expected to cover this time period.

Animal models are essential for the development of new therapies and gaining insight into the pathogenesis of CNV. They are also crucial to refine existing treatments of CNV. Although mimicking the progress and molecular profiles of human CNV, the murine model of laser-induced CNV is not pathogenetically fully consistent with that of wet AMD in human. Secondly, mice do not have a macula, and laser photocoagulation has been used to treat CNV as well as create it [12]. AMD research urgently needs adequate models facilitating investigation of different stages of the disease. Recently, Albert and his co-workers have described CNV formation in a cyclic light induced rat model, where rats were exposed to 12 hours of 3000-lux cyclic light for 1,3 or 6,months. Microscopic sub-RPE NV was observed at 1 month and extension of the NV into the outer retina was detected at 3 months [49]. Featuring the advantage of its potential to study the biologic progression of CNV without laser or mechanical damage to the Bruch's membrane, this rat model exhibits other retinal atrophy and drusen-like deposits induced by cyclic light [12]. Researchers should keep in mind the dynamic processes and involution stages of CNV formation when assessing animal models of CNV. It is almost certain that monotherapy will be replaced by combination therapy, similar to the field of oncology. Although unable to model the complete disease process of human CNV, the animal models will be necessary to investigate the safety and efficacy of combined therapy. Continued research for better, more reliable and reproducible animal models is being carried out.

## Conclusions

16–20-week-old female mice developed the biggest CNV area of those aged 5–8 weeks and 30–40 weeks at the 14^th^ day after laser photocoagulation. Laser spots delivered 1PD away from the optic disc induced bigger area of CNV compared to those 2PD or 3PD away and should be performed in different quadrants of the retina to avoid interference of each other. The best time to observe and analyze is the fourteenth day after photocoagulation.
